# Supplementary material for: Lysine-Specific Demethylase 4D Is Critical for the Regulation of the Cell Cycle and Antioxidant Capacity in Goat Fibroblast Cells
Source: Biology (Basel). 2023 Aug 7;12(8):1095. doi: 10.3390/biology12081095 (PMC10451980; doi:10.3390/biology12081095)
Supplement: Supplementary file 1 [file biology-12-01095-s001.zip › biology-2516297-supplementary.pdf]

Supplementary Materials

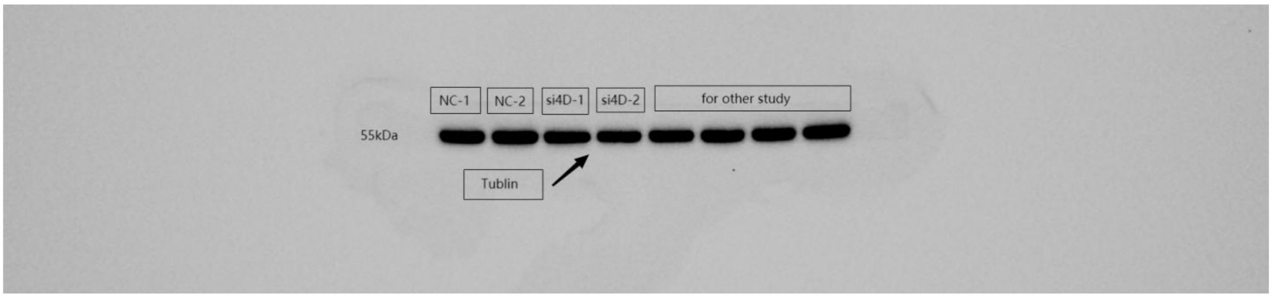

Figure S1. Western blot membrane of Tublin (~55 kDa) protein (loading control) detected with anti-Tublin (66031-1-lg; 1:10000; Proteintech, CHI, USA) antibody. After denaturation, 10 μg of protein samples were loaded and separated by electrophoresis, and transferred to PVDF membranes using a protein rapid transfer apparatus. After blocking in 5% BSA for 2 h at RT, PVDF membranes were incubated with the corresponding primary antibody overnight at 4 °C, then washed in TBST solution and incubated with HRP-goat anti-mouse IgG (SA00001-1; 1:5000; Proteintech, CHI, USA) for 1 h at RT. Subsequently, immunoblotting was visualized using enhanced ECL ultra-sensitive luminescence fluid (32209, Thermo Fisher, Waltham, MA, USA) and exposed with Image Quant LAS 400 (Fiji film, Tokyo, Japan). Fold changes of protein levels were analyzed using Image J software (Wayne Rasband, Bethesda, MD, USA).

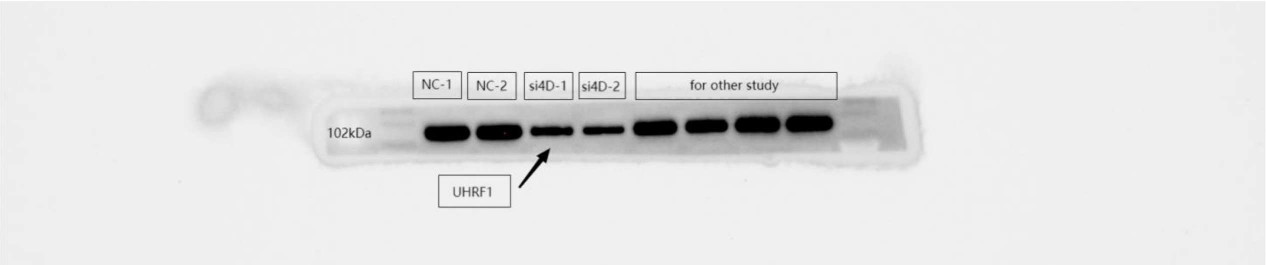

Figure S2. Western blot membrane of UHRF1 (~102 kDa) protein detected with anti-UHRF1 (Ab194236; 1:1000; Abcam, Cambridge, UK) antibody. After denaturation, 10 μg of protein samples were loaded and separated by electrophoresis, and transferred to PVDF membranes using a protein rapid transfer apparatus. After blocking in 5% BSA for 2 h at RT, PVDF membranes were incubated with the corresponding primary antibody overnight at 4 °C, then washed in TBST solution and incubated with HRP-goat anti-rabbit IgG (31460; 1:10000; Pierce, Rockford, USA) for 1 h at RT. Subsequently, immunoblotting was visualized using enhanced ECL ultra-sensitive luminescence fluid (32209, Thermo Fisher, Waltham, MA, USA) and exposed with Image Quant LAS 400 (Fiji film, Tokyo, Japan). Fold changes of protein levels were analyzed using Image J software (Wayne Rasband, Bethesda, MD, USA) and normalized to Tublin.

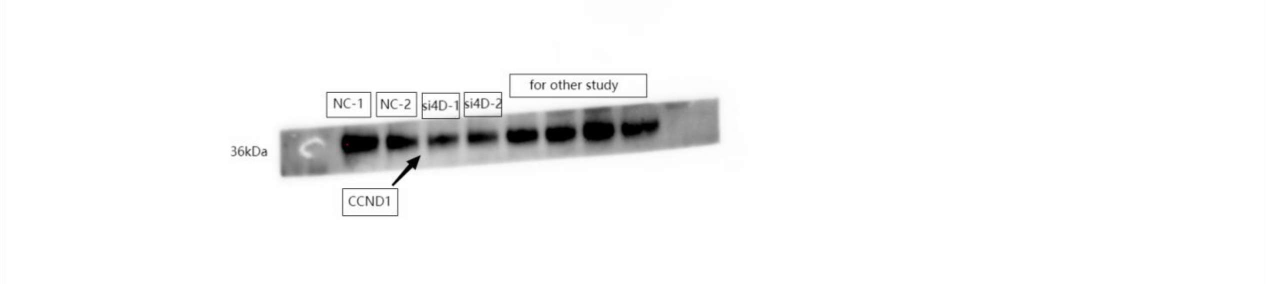

Figure S3. Western blot membrane of CCND1 (~36 kDa) protein detected with anti-CCND1 (60186-1-IG; 1:1000; Proteintech, CHI, USA) antibody. After denaturation, 10 μg of protein samples were loaded and separated by electrophoresis, and transferred to PVDF membranes using a protein rapid transfer apparatus. After blocking in 5% BSA for 2 h at RT, PVDF membranes were incubated with the corresponding primary antibody overnight at 4 ° C, then washed in TBST solution and incubated with HRP-goat anti-mouse IgG (SA00001-1; 1:5000; Proteintech, CHI, USA) for 1 h at RT. Subsequently, immunoblotting was visualized using enhanced ECL ultra-sensitive luminescence fluid (32209, Thermo Fisher, Waltham, MA, USA) and exposed with Image Quant LAS 400 (Fiji film, Tokyo, Japan). Fold changes of protein levels were analyzed using Image J software (Wayne Rasband, Bethesda, MD, USA) and normalized to Tublin.

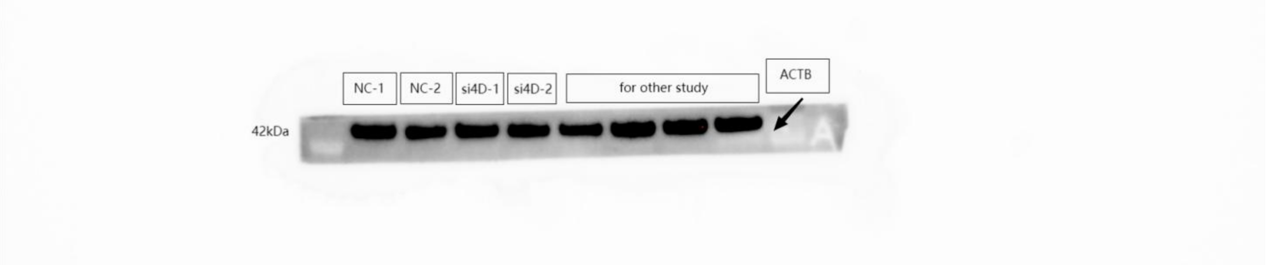

Figure S4. Western blot membrane of ACTB (~42 kDa) protein (loading control) detected with anti-ACTB (Ab8227; 1:4000; Abcam, Cambridge, UK) antibody. After denaturation, 10 μg of protein samples were loaded and separated by electrophoresis, and transferred to PVDF membranes using a protein rapid transfer apparatus. After blocking in 5% BSA for 2 h at RT, PVDF membranes were incubated with the corresponding primary antibody overnight at 4 ° C, then washed in TBST solution and incubated with HRP-goat anti-rabbit IgG (31460; 1:10000; Pierce, Rockford, USA) for 1 h at RT. Subsequently, immunoblotting was visualized using enhanced ECL ultra-sensitive luminescence fluid (32209, Thermo Fisher, Waltham, MA, USA) and exposed with Image Quant LAS 400 (Fiji film, Tokyo, Japan). Fold changes of protein levels were analyzed using Image J software (Wayne Rasband, Bethesda, MD, USA).

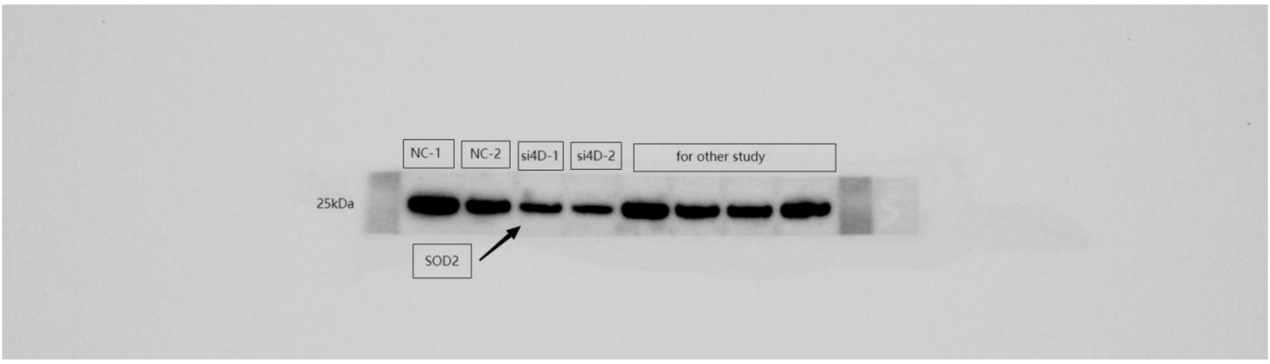

Figure S5. Western blot membrane of SOD2 (~25 kDa) protein detected with anti-SOD2 (24127-1-AP; 1:1000; Proteintech, CHI, USA) antibody. After denaturation, 10 μg of protein samples were loaded and separated by electrophoresis, and transferred to PVDF membranes using a protein rapid transfer apparatus. After blocking in 5% BSA for 2 h at RT, PVDF membranes were incubated with the corresponding primary antibody overnight at 4 ° C, then washed in TBST solution and incubated with HRP-goat anti-rabbit IgG (31460; 1:10000; Pierce, Rockford, USA) for 1 h at RT. Subsequently, immunoblotting was visualized using enhanced ECL ultra-sensitive luminescence fluid (32209, Thermo Fisher, Waltham, MA, USA) and exposed with Image Quant LAS 400 (Fiji film, Tokyo, Japan). Fold changes of protein levels were analyzed using Image J software (Wayne Rasband, Bethesda, MD, USA) and normalized to ACTB.

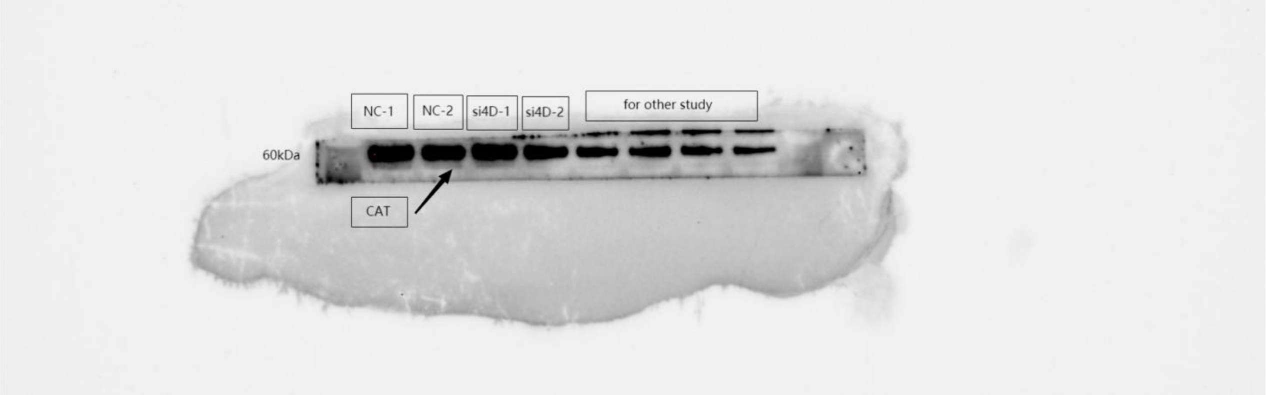

Figure S6. Western blot membrane of CAT (~60 kDa) protein detected with anti-CAT (21260 -1-AP; 1:1000; Proteintech, CHI, USA) antibody. After denaturation, 10 μg of protein samples were loaded and separated by electrophoresis, and transferred to PVDF membranes using a protein rapid transfer apparatus. After blocking in 5% BSA for 2 h at RT, PVDF membranes were incubated with the corresponding primary antibody overnight at 4 ° C, then washed in TBST solution and incubated with HRP-goat anti-rabbit IgG (31460; 1:10000; Pierce, Rockford, USA) for 1 h at RT. Subsequently, immunoblotting was visualized using enhanced ECL ultra-sensitive luminescence fluid (32209, Thermo Fisher, Waltham, MA, USA) and exposed with Image Quant LAS 400 (Fiji film, Tokyo, Japan). Fold changes of protein levels were analyzed using Image J software (Wayne Rasband, Bethesda, MD, USA) and normalized to ACTB.

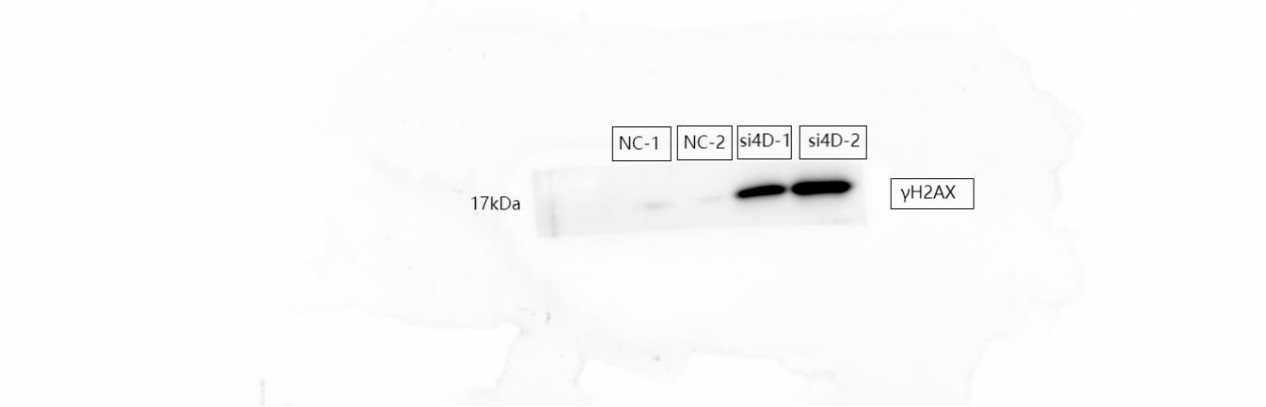

Figure S7. Western blot membrane of γH2AX (~17 kDa) protein detected with anti-H2AX (Ab124781; 1:200; Abcam, Cambridge, UK) antibody. After denaturation, 10 μg of protein samples were loaded and separated by electrophoresis, and transferred to PVDF membranes using a protein rapid transfer apparatus. After blocking in 5% BSA for 2 h at RT, PVDF membranes were incubated with the corresponding primary antibody overnight at 4 ° C, then washed in TBST solution and incubated with HRP-goat anti-rabbit IgG (31460; 1:10000; Pierce, Rockford, USA) for 1 h at RT. Subsequently, immunoblotting was visualized using enhanced ECL ultra-sensitive luminescence fluid (32209, Thermo Fisher, Waltham, MA, USA) and exposed with Image Quant LAS 400 (Fiji film, Tokyo, Japan). Fold changes of protein levels were analyzed using Image J software (Wayne Rasband, Bethesda, MD, USA) and normalized to ACTB.
